# Multi-Host Pathogen *Staphylococcus aureus*—Epidemiology, Drug Resistance and Occurrence in Humans and Animals in Poland

**DOI:** 10.3390/antibiotics12071137

**Published:** 2023-06-30

**Authors:** Aleksandra Trościańczyk, Aneta Nowakiewicz, Martyna Kasela, Anna Malm, Anna Magdalena Tracz, Agata Hahaj-Siembida, Marcelina Osińska, Szczepan Gula, Igor Jankowiak

**Affiliations:** 1 Sub-Department of Veterinary Microbiology, Department of Preclinical Veterinary Sciences, University of Life Sciences in Lublin, Akademicka 12, 20-033 Lublin, Poland; aneta.nowakiewicz@up.lublin.pl (A.N.); anna.tracz@up.lublin.pl (A.M.T.); agata.hahaj-siembida@up.lublin.pl (A.H.-S.); marcelina.osinska@up.lublin.pl (M.O.); 2 Department of Pharmaceutical Microbiology, Medical University of Lublin, Chodzki 1, 20-093 Lublin, Poland; martyna.kasela@umlub.pl (M.K.); anna.malm@umlub.pl (A.M.); 3 Faculty of Veterinary Medicine, University of Life Sciences in Lublin, Akademicka 13, 20-033 Lublin, Poland; szczepangula.p@gmail.com (S.G.); igor16.11.01@interia.eu (I.J.)

**Keywords:** *S. aureus*, MRSA, VISA, MLST, resistance, ADSRRS-fingerprinting

## Abstract

*Staphylococcus aureus* is a drug resistant pathogen with zoonotic potential commonly isolated from humans and animals. The aim of this study was to compare the occurrence of drug resistance, resistance genes, sequence types (STs), and genotypes of *S. aureus* isolated from humans, livestock, and wildlife in eastern Poland. A high percentage of isolates resistant to penicillin (63%), erythromycin (39%), clindamycin (37%), tetracycline (31%), and methicillin (MRSA-19%), an intermediate resistant to vancomycin (VISA-13%), and a multidrug resistant (MDR-39%) was obtained. Multilocus sequence typing analysis showed the presence of 35 different STs (with dominance ST 15, ST 45, ST 7, and ST 582 in human, and ST 398 and ST 8139 in porcine and cattle isolates, respectively), including 9 new ones that had never been reported before (ST 8133-8141). Identical genotypic patterns were detected among porcine and cattle isolates as well as from humans and cattle. A high percentage of MDR, MRSA, and VISA in humans and livestock combined with the presence of the same genotypes among *S. aureus* isolated from human and cattle indicates the circulation of strains common in the region and their zoonotic potential. There is a need to develop new strategies to counteract this phenomenon according to the One Health policy.

## 1. Introduction

One of the key aspects of epidemiology is the assessment of the occurrence and trends in the distribution of microorganisms with particular importance to public health. This group includes antibiotic-resistant *S. aureus* belonging to the ESKAPE group (*Enterococcus faecium*, *Staphylococcus aureus, Klebsiella pneumoniae*, *Acinetobacter baumannii*, *Pseudomonas aeruginosa*, and *Enterobacter* spp.), i.e., it is one of the six pathogens responsible for nosocomial infections [1]. Special attention is directed to methicillin-resistant *S. aureus* (MRSA) due to their insusceptibility to all substances from the β-lactam group on the one hand and the frequent coexistence of multidrug resistance (MDR) and, consequently, the limited therapeutic possibilities on the other hand. Currently, MRSA strains are divided into hospital-associated (HA-MRSA), community-associated MRSA (CA-MRSA), and livestock-associated MRSA (LA-MRSA) [2]. Of particular concern are the increasingly frequent cases of MRSA also occurring in free-living animals, which proves the spread of the phenomenon of drug resistance and suggests the need to include this group of hosts in the drug resistance monitoring [3]. In addition to MRSA, growing attention is also paid to the occurrence of resistance to vancomycin among *S. aureus* (VRSA), and this antibiotic is one of the so-called last-resort drugs for severe MRSA infections [4]. Moreover, the phenomenon of the lack of clinical efficacy in the treatment of *S. aureus* infection, despite the susceptibility of the strains in laboratory tests, has contributed to the introduction of the term vancomycin intermediate *S. aureus* (VISA) referring to strains with a minimum inhibitory concentration (MIC) value of 4–8 µg/mL [5].

The control of the spread of *S. aureus* resistance related to epidemiological analysis is based on various genetic typing techniques. Genetic analyses such as multilocus sequence typing (MLST) or genotyping are particularly useful tools for epidemiological assessment [6,7]. Comparative analysis of sequence types or genotypic patterns of strains characterized by resistance to key drugs in medicine makes it possible to predict resistance distribution trends. It is believed that certain sequence types/clonal complexes (STs/CCs) are correlated with particular groups of MRSA (e.g., ST 5, ST 22 with HA-MRSA, ST 1 with CA-MRSA, or ST 398 with LA-MRSA) [8]. However, cases of isolation of *S. aureus* with sequence types atypical for given hosts/environments are increasingly being observed, which proves the adaptability of the species to inhabit various niches [9]. Given the increasing prevalence of drug resistance, including multidrug resistance, the transfer of *S. aureus* between hosts representing different species is of particular concern. Previous studies more and more often indicate the possibility of transferring zoonotic *S. aureus* to humans, and vice versa but the knowledge of the trends in the distribution of this pathogen needs to be supplemented and expanded [9]. The comparative analysis of the occurrence of drug resistance, STs, and genotypes of *S. aureus* isolated from different groups of hosts (humans, farm animals, and wildlife) from a common geographical region presented in this paper may be a valuable contribution to the supplementation of this information.

The aim of this study was to carry out a comparative analysis of drug resistance profiles and the occurrence of drug resistance genes, sequence types, and genotypes of *S. aureus* isolated from various hosts (human, pigs, cattle, and wildlife) from eastern Poland in the aspect of the zoonotic potential of these bacteria.

## 2. Results

The occurrence and percentage of *S. aureus* isolation from the individual farms are presented in Table 1. Of the 248 pig nasal swabs, 21% were positive for *S. aureus* (51 isolates from 3 farms). The percentage of isolation of *S. aureus* was 5% from the bovine samples (36/680 from 11 farms) and 13% (12/113) from the wildlife. *S. aureus* were isolated from a total of 10 people involved in animal care (1 pig farm (*n* = 2) and 6 cattle farms (*n* = 8)), which accounted for 67% (10/15). No *S. aureus* were isolated simultaneously from the animals and their keepers on any pig farm (two *S. aureus* strains were isolated only from the humans on farm R and only from the pigs on farms N, O, and P). In the case of 4 cattle farms (B, C, G, I), *S. aureus* were isolated from both animals and animal owners. Three *S. aureus* strains were isolated from the animal keepers on 2 cattle farms (D (*n* = 2) and J (*n* = 1)), where none of the cows were carriers of *S. aureus.*

All isolates were tested for susceptibility to penicillin (PEN), cefoxitin (FOX), gentamicin (GEN), tetracycline (TET), erythromycin (ERY), clindamycin (CLI), quinupristin-dalfopristin (QD), ciprofloxacin (CIP), enrofloxacin (ENR), chloramphenicol (CHL), trimethoprim-sulfamethoxazole (SXT), rifampin (RIF), nitrofurantoin (NIT), linezolid (LZD), and vancomycin (VAN). The *S. aureus* strains (*n* = 239) exhibited the highest percentage of resistance to PEN (63%), ERY (39%), CLI (37%), and TET (31%) and the lowest resistance to RIF and LZD (0.4% for both) (Table 2). In the case of CLI, inducible resistance to this agent (CLIind.) was detected in 3% (7/239) of *S. aureus* (all isolated from humans). The *S. aureus* drug susceptibility studies revealed a relatively high percentage of MRSA (19%; 46/239) and VISA (13%; 30/239) (Table 2). The occurrence of resistance to the tested antibacterials varied depending on the host group, and there were statistically significant differences in the level of resistance to the antibacterial substances, depending on the host. Compared to the isolates from the other hosts, *S. aureus* isolated from the pigs were characterized by a higher percentage of resistance to CLI (100%; 51/51), TET (94%; 48/51), FOX (76%; 39/51), and ERY (86%; 44/51) (*p* < 0.05) (Table 2). A high percentage of VISA (14%; 5/36) was found in the case of *S. aureus* isolated from the cattle. The lowest percentage of *S. aureus* resistance to the tested agents was detected in the wildlife hosts. *S. aureus* strains with resistance to penicillin (17%; 2/12), tetracycline (8%; 1/12), and gentamicin (8%; 1/12) were found in these hosts. *S. aureus* isolated from the humans were characterized by a high level of resistance to PEN (69%; 96/140), ERY (35%; 49/140), CLI (CLI—19%; 26/140 + CLIind.—7%; 7/140), CIP (19%; 26/140), and VISA (18%; 25/140) (Table 2).

The present study showed a high diversity in the phenotypic antimicrobial resistance profiles of *S. aureus* (Table 3). There were 47, 13, 6, and 3 resistance profiles observed in the *S. aureus* isolated from the humans, pigs, cattle, and wildlife hosts, respectively. PEN was the dominant resistance profile of *S. aureus* isolated from the humans (*n* = 28). PEN, FOX, CLI, TET, and ERY were the most common resistance profiles of *S. aureus* isolated from the pigs (*n* = 36). This profile was obtained from all the isolates from farm P. The resistance profiles of *S. aureus* isolated from the animals on the other pig farms showed variability (farm N and O: 8 and 3 resistance profiles, respectively). VAN was the dominant resistance profile (*n* = 5) of *S. aureus* isolated from the cattle. No identical resistance profiles of *S. aureus* isolated from the farm animals and humans taking care of these animals were found. In the case of *S. aureus* isolated from the wildlife, three strains with the following resistance profiles were detected: PEN, GEN and PEN, and TET. Fifty-eight *S. aureus* (24%) showed no resistance to any of the tested drugs. The percentage of *S. aureus* with susceptibility to all the tested antibacterial substances isolated from the individual human, pig, cattle, and wildlife hosts was 17% (24/140), 0%, 69% (25/36), and 75% (9/12), respectively (Table 3).

Based on the analysis of the drug resistance of all the isolated *S. aureus*, the prevalence of the MDR isolates was 39% (94/239). The highest MDR rates were found among *S. aureus* isolated from the pigs (98%; 50/51), human carriers (40%; 4/10), and hospitalized individuals (30%; 39/130). MDR was not found among the wildlife isolates (Table 4). Among MDR *S. aureus*, the largest group comprised isolates characterized by a lack of resistance to five tested antibacterial substances. Furthermore, two human isolates were found to be resistant to nine and eleven drugs, respectively (Table 4).

Twelve of the sixteen tested antimicrobial resistance genes were detected among the *S. aureus* isolates. None of the isolates showed the presence of the *van*A and/or *van*B genes. The results regarding the occurrence of individual resistance genes and their distribution are presented in Table 5 and Table 6, respectively. Regardless of the host group, *bla*Z was the most commonly detected gene (59%; 141/239). Moreover, 29% (44/150) of PEN-resistant *S. aureus* simultaneously possessed *bla*Z, *mec*A, and *mec*C genes. Ninety-four percent (141/150) of the isolates with phenotypic resistance to PEN carried the *bla*Z gene. Of the 46 MRSA, 45 were characterized by the simultaneous presence of *mec*A and *mec*C genes. The phenotypic resistance to GEN was confirmed by the detection of the *aac(6′)-Ie-aph(2″)-Ia* gene in 61% of *S. aureus* (11/18). The presence of *tet*M and *tet*K encoding resistance to TET was observed in 31% and 23% of all *S. aureus*, respectively (i.e., in 74 out of the 75 *S. aureus* isolates resistant to this antibiotic). Seventy-five percent (56/75) of isolates with phenotypic TET resistance carried both genes. In the present studies, the phenotypic resistance to ERY was associated with the presence of four genes (*erm*A, *erm*B, *erm*C, and *msr*A), with *erm*B detected most frequently. Moreover, 49% (46/94) of the ERY resistant isolates had two genes, of which the most common configuration was *erm*A and *erm*B (45%; 46/94), and 2% (2/94) were carriers of all four tested genes. Of the 81 CLI-resistant isolates, 52% (*n* = 85) were carriers of 2 resistance genes, with *erm*A and *erm*B (49% of all isolates resistant to this drug; 44/85) as the dominant combination; in turn, none of the *erm* genes were found in 21% (18/85). Half of the QD-resistant isolates had at least one gene conferring resistance to streptogramin. Among these isolates, *erm*C was the most frequently detected gene. In the group of the CHL-resistant isolates, 57% (4/7) were carriers of *cat* (pC221) and 14% (1/7) carried *cat* (pC223). There were no isolates carrying both CHL resistance genes and the *cat* (pC194) gene. Statistically higher differences in the percentage of detection of the *bla*Z, *mec*A, *mec*C, *tet*M, *tet*K, *erm*A, and *erm*B genes were observed between porcine *S. aureus* isolates and those isolated from the other hosts.

**Figure 1:**
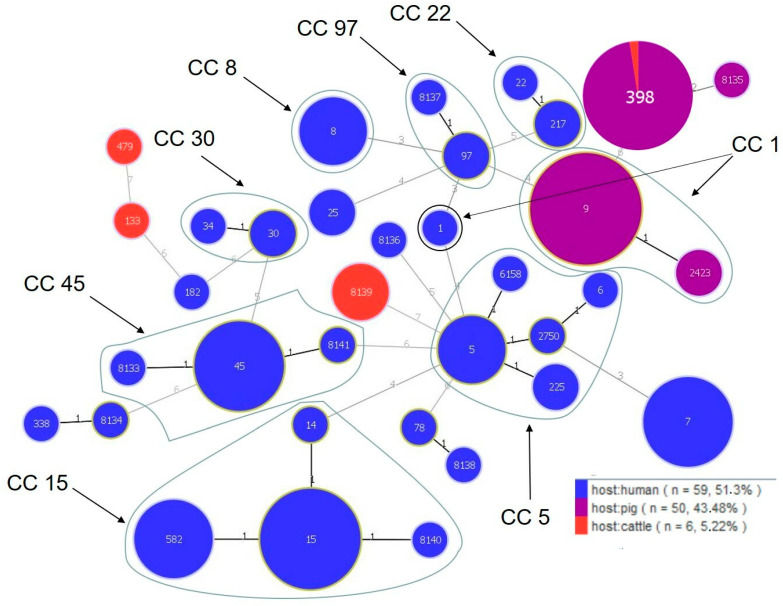
Population structure of *S. aureus*. Each ST is represented by a dot. Numbers on the lines connecting STs indicate locus differences.

Based on the MLST analysis, the MDR and VISA isolates were classified into 35 different STs, including 9 new ones that had not been previously reported (ST 8133-8141) (Figure 1). New STs were identified in *S. aureus* isolated from the humans (ST 8133, 8134, 8136, 8137, 8138, 8140, and 8141), pigs (ST 8135), and cattle (ST 8139). The highest variation in STs was exhibited by the human isolates (28 different STs), with ST 15 (*n* = 7), ST 45 (*n* = 6), ST 7 (*n* = 6), and ST 582 (*n* = 5) as the most common types (Table 7). The isolates from the pigs and cattle represented four different STs each. The dominant STs in the *S. aureus* isolates from the pigs and cattle were ST 398 (*n* = 39) and ST 8139 (*n* = 3), respectively (Table 7). There were also differences in the occurrence of STs within a single farm. In the case of *S. aureus* isolated from the pigs on farm N, a variation in the occurrence of STs (three different STs) was observed, in contrast to the isolates from farm P (*n* = 36) representing the same ST 398. No identical STs were found among *S. aureus* isolated from the livestock and the human keepers of these animals. Only one ST was found among the isolates from two different host species (ST 398 was found among 39 *S. aureus* isolates from the pigs (farm O *n* = 3, farm P *n* = 36) and one isolated from the cattle) (Table 7). The *S. aureus* isolates were classified into eight clonal complexes (CC1 included ST 1, 9, and 2423; CC5 included ST 5, 6, 225, 2750, and 6158; CC8 included ST8; CC15 included ST 14, 15, 582, and the new complex, 8140; CC22 included ST 22 and 217; CC30 included ST 30 and 34; CC45 included ST 45 and two new complexes, ST 8133 and 8141; CC97 included ST 97 and the new complex, 8137) (Table 7). Most isolates whose STs were assigned with CCs were of human origin. Only CC1 comprised *S. aureus* isolated from both humans and pigs, but belonging to different STs (Table 7, Figure 1). The GoeBURST analysis based on single locus variants (SLVs) showed the presence of 9 complexes, including 7 classified as CCs (CC1, 5, 15, 22, 30, 45, and 97), and 13 singletons (Figure 1).

**Figure 2:**
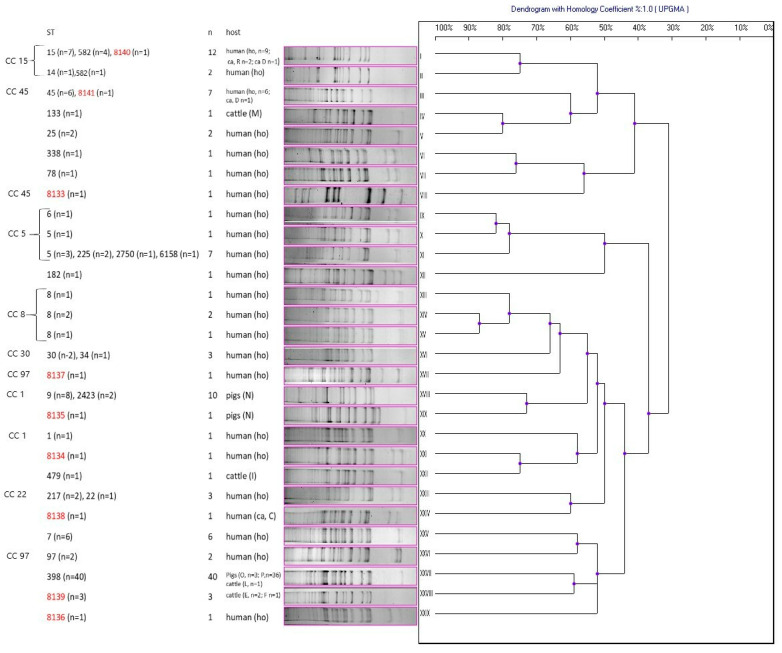
Dendrogram of similarity of multidrug-resistant and vancomycin intermediate susceptible *S. aureus* (MDR/VISA) based on ADSRRS-fingerprinting results; I–XXIX—clusters; the new ST is marked in red; R, D, M, N, I, C, O, P, L, E, F—farm markings; ho—hospitalized patients; ca—carriers.

**Figure 3:**
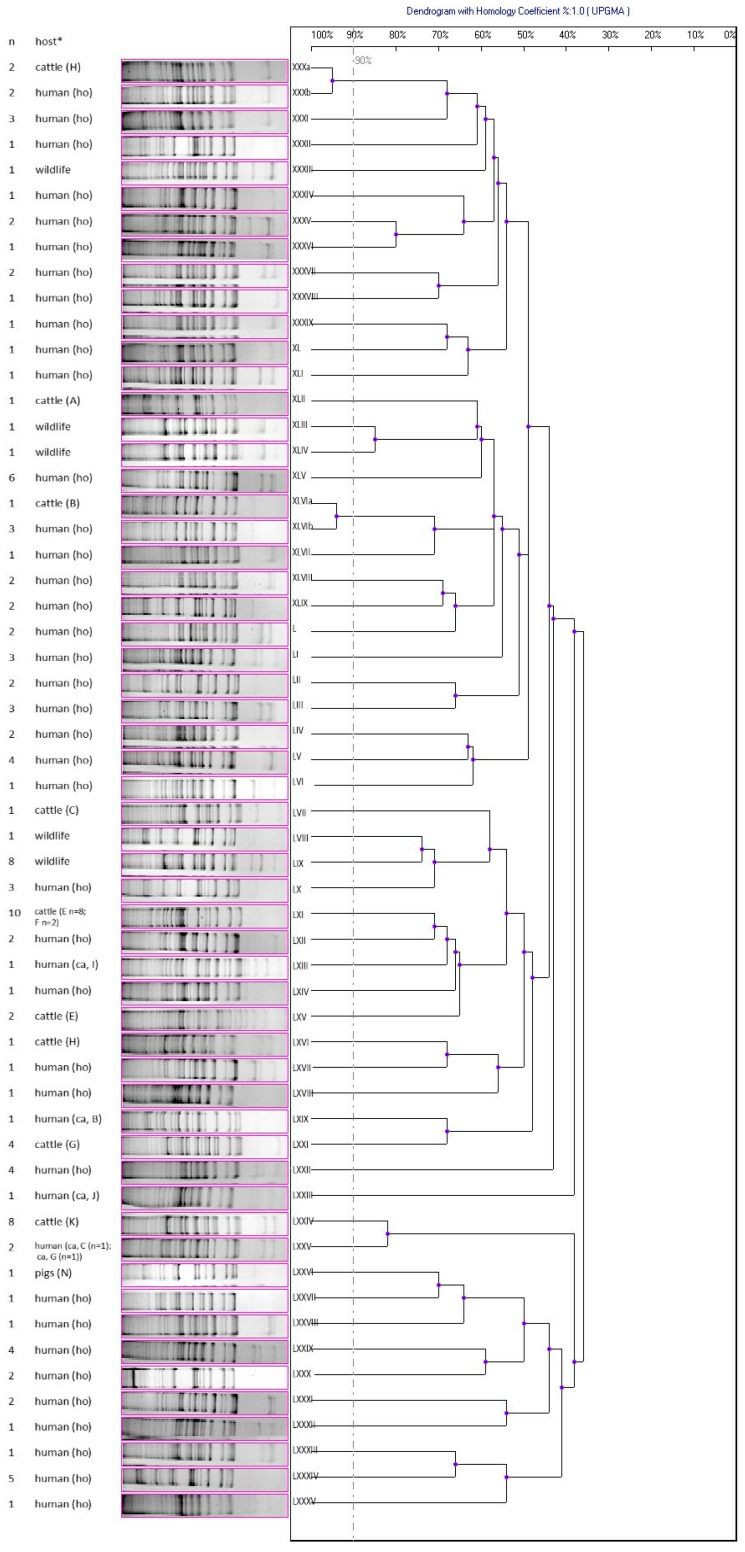
Dendrogram of similarity of non-multidrug-resistant and non-vancomycin intermediate susceptible *S. aureus* isolates (non-MDR/VISA) based on ADSRRS-fingerprinting results; XX–LXXXV—clusters; * H, A, B, C, E, F, I, G, J, K, N—farm markings; ho—hospitalized patients; ca—carriers.

Amplification of DNA fragments surrounding rare restriction sites (ADSRRS-fingerprinting) was performed for all isolates. The obtained electrophoretic profiles of the MDR/VISA isolates are shown in Figure 2 and the other ones are presented in Figure 3. The analysis of the MDR/VISA isolates carried out in these studies allowed identification of 29 different genotypic profiles (I-XXIX), with the most numerous genotypes being XXVII (*n* = 40), I (*n* = 12), and XVIII (*n* = 10) (Figure 2). For the MDR/VISA isolates of human, bovine, and porcine origin, 23, 4, and 3 distinct genotypic profiles were obtained, respectively. In one case, the same genotypic profile (XXVII) characterized *S. aureus* from two different host species (pigs and cattle). Moreover, the same genotypic profiles (I and III) were obtained for the MDR/VISA isolates from both hospitalized patients and human carriers. The dendrogram obtained on the basis of the ADSRRS-fingerprinting results shows high similarity (70–100%) of isolates within some CCs (CC15, 5, 8, 30, and 22). Most isolates representing different STs but classified to the same CC were characterized by an identical genotypic profile (I, II, III, XI, XVI, XVIII, and XXIII). On the other hand, there were differences in the genotypic profile between some isolates classified under the same ST (e.g., profile I and II grouped isolates belonging to ST 582) (Figure 2). No relationship was observed between the resistance profile/presence of resistance genes and the genotype (Table 7).

Based on the results of the ADSRRS-fingerprinting genotyping of the non-MDR/VISA isolates, a dendrogram was obtained containing 56 different genotypic profiles, among which the most numerous profiles were LXI (*n* = 10), which characterized the bovine isolates, LIX (*n* = 8) grouping the wildlife isolates, and LXXIV (*n* = 8), which included the bovine isolates (Figure 3). In the case of the free-living animals, five different genotypic profiles were observed, with the dominance of the LIX profile. In two cases, the same genotypic profiles (>90% similarity) were detected in the human (hospitalized) and bovine *S. aureus* isolates (profile XXX and XLVI) (Figure 3).

In the analysis of the dendrograms of both MDR/VISA and other isolates, the same genotypic profiles were not found among *S. aureus* isolated from the farm animals and the owners/keepers of these animals (Figure 2 and Figure 3). On the one hand, different genotypic profiles were observed among *S. aureus* isolated from the livestock on one farm, e.g., H (two different profiles), E (three different profiles), F (two different profiles), and N (three different profiles); on the other hand, some farms were characterized by the presence of *S. aureus* belonging to one genotyping profile (e.g., farm P, K) (Figure 2 and Figure 3).

## 3. Discussion

One Health is a multifaceted and balanced interdisciplinary approach to public health as an interdependent correlation between people, animals, and the shared environment [10]. The key aspects within One Health are the monitoring of the spread of drug resistance and the control of pathogens with zoonotic potential [10]. One pathogen that shares both those features is *S. aureus.* Research on the characteristics of *S. aureus* isolated from various host species, including humans, livestock, and free-living animals, can be a valuable contribution to the protection of public health under One Health.

One of the biggest global problems is the growing phenomenon of drug resistance among bacteria that are crucial to public health. The occurrence of MDR *S. aureus* has been recorded in Poland among humans, livestock, and free-living animals [3,11,12]. Due to the interpenetration of the living environments of these hosts, there is a risk of interspecies transmission of these dangerous pathogens [13]. The occurrence of drug resistance in bacteria, including MDR, is directly related to previous exposure to antibacterial substances [14]. Due to some differences in the use of antimicrobial agents in the treatment of humans and livestock, the resistance patterns of *S. aureus* in these host groups may differ. However, a β-lactam antibiotic penicillin is one of the first-line drugs used to treat *S. aureus* in both groups. The occurrence of penicillin-resistant *S. aureus* was first reported in the 1940s and was related to the hospital environment [15]. Our studies showed the presence of penicillin-resistant isolates among *S. aureus* isolated from all host groups, including the wildlife, with the highest penicillin resistance percentage among the porcine isolates (96%). Our research shows a tendency for penicillin-resistant strains of *S. aureus* to also spread in previously untreated wild animals, which confirms the common resistance to this drug. This phenomenon may result from the synanthropization of these groups of animals in response to changes in the environment introduced by humans [16]. Previous studies have shown that the diversity of antimicrobial resistance genes is one of the adaptive elements of *S. aureus* to a specific host, e.g., the more frequent occurrence of the *bla*Z gene (encoding β-lactamase) in *S. aureus* isolated from humans [17]. In this study, the rate of *bla*Z detection among porcine *S. aureus* was higher than in the human isolates, which is most likely related to the relatively frequent use of penicillin in pig production in Europe [18]. Moreover, in this study, the presence of *bla*Z was also found in the isolates from the cattle and roe deer. Studies conducted by other authors show equally frequent detection of the *bla*Z gene among *S. aureus* isolated from pigs [19], other food animals [20], and wildlife [21], which indicates a relatively wide spread of *S. aureus* carriers of this gene also among hosts other than humans.

The occurrence and characteristics of MRSA are one of the key issues of modern epidemiology. The present study showed differences in the occurrence of MRSA depending on the group of hosts. A relatively high percentage of MRSA (with detection of *mec*A and *mec*C genes) was detected in the porcine isolates (76%), compared to studies published by other scientists from Poland in 2017, where this percentage was 38% [12]. This seems particularly worrying, as the results in the current study showed a two-fold increase in the MRSA carrier rate in pigs. Moreover, our research showed a slight increase in the percentage of MRSA isolation among hospitalized patients (4.6%) compared to earlier studies, where the percentage of MRSA isolation in Lublin province was 2.7% [11]. In contrast to the studies conducted by Mroczkowska et al. [12], the present investigations did not show the prevalence of MRSA among livestock owners. Cases of MRSA occurrence in cattle in Poland have been recorded relatively recently (2020) [22]. While these previous studies were carried out on cow milk samples, our study revealed for the first time in Poland the presence of MRSA in the nasal cavity of cattle, which proves the ability of MRSA to colonize various niches and indicates an increased risk of transmission of the strain between individuals.

Previous studies conducted in Poland on material collected from free-living animals suggest that single animals from the wildlife group may also be a reservoir of MRSA [3]; however, this has not been confirmed in our research. The lack of carrier status in free-living animals in the present study may be related to the species and type of diet (herbivores) of animals from which the material was derived. Predatory animals are much more likely to carry MRSA strains due to their more varied diets compared to herbivores.

One method of treating MRSA infection in humans is the use of a glycopeptide antibiotic vancomycin as a last-resort drug. However, the occurrence of VISA strains, which are the cause of failures in the use of vancomycin in treatment, is increasingly being noted [23]. In this study, a relatively high percentage of VISA (13%) was exhibited by *S. aureus* isolated from humans (18%, hospitalized and livestock farmers) and cattle (14%). There is little data on the occurrence of VISA in humans in Poland. In comparison with the results obtained by Młynarczyk et al. in 2002 (VISA 0.3%) [24], our study showed a relatively high percentage of VISA isolation from humans (both hospital patients and farmers), which suggests that the problem has been growing over the years. Taking into account the limited number of publications on the occurrence of VISA from various hosts in Poland (e.g., no data on the occurrence of VISA in livestock or free-living animals) and the high percentage of VISA occurrence in humans and cattle confirmed by our research, it seems that the problem of the lack of susceptibility of *S. aureus* to glycopeptides may be an underestimated phenomenon in Poland and requires more attention.

Due to the synergistic effect of aminoglycosides with drugs acting on the cell wall (e.g., β-lactams), the occurrence of aminoglycoside-resistant *S. aureus* is a serious problem, especially in treatment of MDR pathogens [25]. Our research showed the presence of GEN-resistant *S. aureus* in humans, pigs, and wildlife. Aminoglycosides are currently in fifth place among the groups of antibacterial substances used in the treatment of food-producing animals [18]. Moreover, the frequency of GEN use in human medicine is high [26]. These are most likely the main causes of the occurrence of resistance to aminoglycosides among *S. aureus* isolated from the humans and livestock observed in our study and reported by other researchers [27]. The interpenetration of human and animal environments, including wild animals, is conducive to the spread of resistant strains [26], which may explain the occurrence of resistance to GEN also among isolates from wildlife. One of the most common mechanisms of resistance to aminoglycoside antibiotics is the synthesis of the enzyme acetyltransferase/phosphotransferase AAC(6′)-Ie/APH(2″)-Ia (encoded by the *aac(6′)-Ie-aph(2″)-Ia* gene) [28]. In our studies, the presence of this gene was detected among the human and porcine isolates. The absence of this gene among some of the isolates showing phenotypic resistance to GEN, including wildlife, suggests the presence of another mechanism of resistance [26].

In our studies, TET was the antibacterial substance with one of the highest percentages of resistant isolates. Moreover, TET resistance was found in *S. aureus* isolated from all the host groups. A particularly high percentage of TET-resistant *S. aureus* was detected among the porcine isolates (statistically significant differences), which is probably a result of the large-scale use of this antibiotic in animal production [18]. The phenotypic resistance to TET in our studies was confirmed by the detection of the *tet*M and *tet*K genes, i.e., most frequent determinants of the occurrence of *S. aureus* resistance to this drug [29]. The high proportion of *S. aureus* isolates with both genes in our study demonstrates a dual “defence strategy” against TET of these microbes, i.e., active protection of the ribosome (*tet*M) and active efflux (*tet*K) [29], which additionally influences the spread of the phenomenon of resistance to this antibiotic.

Our studies showed a relatively high degree of resistance of *S. aureus* to ERY, CLI, and QD, especially among the porcine isolates (86, 100, and 18%, respectively), which is probably the result of a relatively high percentage of use of macrolides and lincosamides in the treatment of farm animals in Europe, especially pigs (8.5% and 4.7%, respectively) [18]. The mechanisms of resistance to macrolide, lincosamide, and streptogramin B (MLS_B_) antibiotics involve modification of the target site (*erm* gene–MLS_B_), active expulsion by the efflux pump (*msr* gene–MS_B_), and drug inactivation (enzymes encoded by genes specific to particular types of resistance) [30]. This study showed that 78% of the erythromycin-resistant *S. aureus* isolates carried at least one macrolide resistance gene, with the *erm*B gene noted most frequently, in contrast to previous studies indicating *erm*A and *erm*C as the most common genes reported among MLS_B_ *S. aureus* [31,32]. The presence of more than one resistance gene noted in our study among *S. aureus* with the MLS_B_ phenotype (49% and 2% of *S. aureus* were carriers of two and four macrolide resistance genes, respectively) indicates various ways of adaptation of this microorganism to unfavourable factors and selection pressure of resistant strains. Considering the ubiquitous presence of macrolide resistance genes among the *S. aureus* isolates observed in this study, the presence of the same resistance determinants in other microorganisms of key importance to public health, i.e., *Enterococcus*, *Streptococcus*, and *Pseudomonas*, and the possibility of gene transfer both within and between species by localization of genes on genetic elements, the spread of drug resistance is a serious public health problem [33]. Furthermore, induced resistance to clindamycin has also been found in the human isolates in this study. While constitutive resistance to clindamycin can be detected by the disc diffusion method routinely used in laboratories, the D-test for detection of induced clindamycin resistance is rarely used. This may lead to an erroneous belief about the susceptibility of some strains to this drug and, consequently, to ineffective treatment [34].

In this study, we observed a relatively high level of resistance to fluoroquinolones (CIP 19% and ENR 9%) among the human *S. aureus* isolates, which confirms the previous report on *S. aureus* strains in Polish hospitals (resistance to ciprofloxacin in MRSA—58%, non-MRSA—1.4%) [11]. Although ENR is not used in human medicine, our research showed the presence of *S. aureus* strains with resistance to this drug. This phenomenon, also observed among *E. faecium* isolates, is most likely related to the phenomenon of cross-resistance to drugs from the group of fluoroquinolones [35].

Despite the limitations of use of CHL in human medicine (treatment of eye infections, otitis externa, severe brain infections, and adverse effects), resistance to this drug among *S. aureus* isolated from humans in Poland is not uncommon (resistance to CHL in MRSA and non-MRSA: 12.8% and 4.6%, respectively) [11], which was also confirmed by our research (5% of CHL-resistant *S. aureus* isolated from the humans). The absence of CHL resistance in *S. aureus* isolated from the animals in this study results from the prohibition of the use of this drug in food-producing animals [36]. One mechanism of resistance to CHL is the production of acetyltransferases (CAT) [28]. In our studies, the presence of *cat* genes (pC221 or pC223) was detected in 71% of CHL-resistant isolates. The frequent detection of *cat* genes confirmed in this study combined with their localization on plasmids suggests the ease of the spread of resistance to CHL between isolates.

The MLST technique is one of the basic epidemiological tools for assessing the occurrence and trends in the distribution of species, particularly those that pose a threat to public health, including MDR *S. aureus.* Previous epidemiological studies of *S. aureus* strains indicated species specificity of STs and CC [8,12,37]. The present study showed the dominance of some STs and CCs among the human isolates (hospitalized patients and carriers), such as CC15, CC45, CC5, CC1, CC8, and CC30, i.e., typical human clones, which is consistent with global data [38]. However, recent reports indicate that some STs of *S. aureus* with a specificity for animal hosts are increasingly being observed among hosts belonging to other species, including humans [9]. An example of this phenomenon is the detection of CC97 in this study, i.e., a clonal complex associated with livestock (ruminants) [39], in the hospitalized patients (*n* = 3). In recent years, increasingly frequent cases of CC97 have been observed in the hospital environment, in people unrelated to farm animals, as well as in Poland, which proves that the differences in the occurrence of specific STs and CCs in individual hosts are slowly blurring [40,41]. Current research also focuses on the spread of MRSA ST 398, which is the most frequently reported sequence type in porcine isolates of *S. aureus*. The presence of this type is also increasingly being found in other animal species and humans, especially those in contact with pigs, suggesting the ease of spread by a direct route of transmission [42]. Moreover, the increasing occurrence of ST 398 in hospitals proves its high epizootic potential, which is a worrying phenomenon in terms of public health protection [43]. This study showed a significant dominance of ST 398 among the porcine isolates (*n* = 39), confirming the results of previous studies conducted in this host group in Poland [12]. What is more, in our study, ST 398 was also detected in one bovine isolate. Since the first cases of the presence of ST 398 *S. aureus* isolated from cattle in Poland were recorded in 2020 [22], our research confirms the tendency of this ST to spread also among hosts other than pigs. While ST 398 was isolated from milk in the aforementioned study, our research showed for the first time the presence of this ST in the nasal cavity of a cow in Poland, which proves the ease of colonization and spread. In contrast to the previously reported cases of ST 398 among people working with animals in Poland (farmers and veterinarians) [12], this study did not show the presence of this lineage in the *S. aureus* isolated from the humans. This was undoubtedly associated with the lack of animal owners’ consent for taking swabs on farms where the presence of ST 398 was detected in the porcine and bovine isolates (farm O, P, and L). Moreover, despite the increasing number of cases of ST 398 isolation from the hospital environment worldwide [43], our research conducted in Poland did not confirm the occurrence of ST 398 among *S. aureus* isolated from the hospitalized patients.

In this study, nine new STs were noted, including four classified as CCs. All isolates classified into the new sequence types identified in this study were multidrug resistant or VAN resistant. Moreover, three bovine *S. aureus* isolates classified to ST 8139 and having the same genotypic pattern and resistance profile (VAN) were isolated from two different farms, suggesting the ease of spread of some STs between farms. The occurrence of allele changes resulting in the formation of new STs within CCs with public health relevance (such as CC15, CC45, and CC97) coupled with the emergence of multidrug resistance, including methicillin resistance, is a major challenge for epidemiological research focused on the control of the spread of *S. aureus*.

In addition to MLST, genotyping techniques based on ligation of oligonucleotide adapters are an effective tool in molecular epidemiology [7]. Another useful tool is the ADSRRS-fingerprinting method. Due to its high differentiating potential, this technique is comparable to pulsed-field gel electrophoresis (PFGE) and has been successfully used in epidemiological studies of *S. aureus* [7]. This study of MDR *S. aureus* showed differences in the genotypic profiles within the individual host groups, which mostly correlated with STs and/or CCs. The presence of different genotypic patterns of isolates belonging to the same ST observed in the present study may result from their genome plasticity, adaptive mechanisms, mutations, or even the influence of the host genotype [37,44]. Since MLST is based on the analysis of housekeeping genes, it detects changes that accumulate slowly in the genome [45]. On the other hand, such fingerprinting techniques as ADSRRS used in this study reflect changes resulting from horizontal gene transfer or mutations, which make them more useful for short-term epidemiological studies [7]. For these reasons, it seems necessary to use several tools for a complete epidemiological analysis.

Cases of interspecies transmission of *S. aureus* strains have been confirmed in various studies [22,42]. In our study, we showed the occurrence of the same pattern obtained after ADSRRS-fingerprinting among isolates belonging to pigs and cattle in the MDR *S. aureus* group as for the human and bovine isolates in the non-MDR group. The presence of the same genotypic profiles of *S. aureus* isolated from the humans (hospitalized) and the animals obtained in our study, in the absence of isolates with identical genotypic profiles isolated from the humans directly involved in the care of the animals and these animals, suggests other possibilities of pathogen transmission than direct contact. Due to the ability of *S. aureus* to survive in a dry environment and the presence of these bacteria in dust, the role of airborne transmission in the spread of this pathogen is increasingly being indicated [46].

The present study showed a diversity of antimicrobial resistance profiles, resistance genes, genotypic patterns, and STs of *S. aureus* isolated on one farm on the one hand; on the other hand, the investigations showed the occurrence of *S. aureus* with identical characteristics isolated from animals on the same farm, suggesting the dominance of one strain in the colonization of hosts of the same species living in a common space (e.g., farm P). The probable colonization with one strain (the same STs and genotype profiles) of a large number of animals (farm P, *n* = 36) in connection with its multidrug resistance (resistance to 5 drugs) seems particularly disturbing. This trend combined with the possibility of colonization of different host species (including humans) by the same strains, which was also found in the present study, suggests the need for constant epidemiological monitoring of *S. aureus* in different hosts that can be a potential source of pathogens posing health risks to humans.

The present investigations are the first such extensive studies on the characteristics of *S. aureus* in Poland in terms of carrier status in various host groups. They indicate not only a progressive increase in the drug resistance of these bacteria, including methicillin resistance and multidrug resistance, but also the adaptation of the strains to different ecological niches, and thus the possibility of transmission between different host species, including humans.

## 4. Materials and Methods

### 4.1. Bacterial Strains

The research material consisted of 130 *S. aureus* strains from the collection of the Department of Pharmaceutical Microbiology, isolated from skin and soft tissue infections from hospitalized patients, 248 pig nasal swabs from 8 farms, 3 nasal swabs from animal keepers on 2 pig farms, 680 cattle nasal swabs from 25 farms, 12 nasal swabs from animal keepers on 10 cattle farms and 113 nasal swabs from wildlife (*Capreolus capreolus* and *Cervus elaphus*). The number of swabs taken from the hosts on each farm is shown in Table 8. Human-derived material was collected with prior personal consent.

Material from wildlife came from animals culled by authorized hunters in hunting districts in eastern Poland in accordance with the regulations of the Hunting Law Act of 20 May 2020 (Dz. U. [*Journal of Laws*] 2020 no. 67, items 148, 695, and 875.) and the Regulation of the Minister of Climate and Environment of 29 June 2022 amending the regulation on determining hunting seasons for game animals. The samples came from dead free-living animals; hence, the approval of the ethics committee was not required.

Samples were collected with a sterile cotton swab with Amies medium (Medlab-Products, Raszyn, Poland), transported under refrigeration conditions for a maximum of 3 h, and stored at −80 °C for further analysis. The swabs were pre-incubated in buffered peptone water (37 °C, 24 h) and then plated on a Baird Parker Lab-Agar (Biomaxima, Lublin, Poland). After incubation (37 °C for 24 h), one typical black colony from each sample was further analyzed (Gram stain and coagulase test). Species identification was based on the protocol described by Sasaki et al. [47]. DNA was isolated using the Gram Plus & Yeast Genomic DNA Purification Kit (EUR_x,_ Gdańsk, Poland) according to the manufacture’s procedure.

### 4.2. Evaluation of Drug Resistance

The drug resistance of the strains was determined with the disc diffusion method in relation to PEN 10 U, FOX 30 µg, GEN 10 µg, TET 30 µg, ERY 15 µg, CLI 2 µg, QD 15 µg, CIP 5 µg, ENR 5 µg, CHL 30 µg, SXT 1.25/23.75 µg, RIF 5 µg, NIT 300 µg, and LZD 30 µg (Oxoid, Hampshire, UK) and with the broth microdilution method in relation to VAN 0.25–128 µg/mL (Sigma-Aldrich, Darmstadt, Germany) according to the Clinical and Laboratory Standards Institute (CLSI) criteria [48,49]. The presence of inducible clindamycin resistance (ICR) was checked by the D-zone test. The classification of the MDR strains was based on the criteria described by Magiorakos et al. (lack of susceptibility to at least one agent from three or more classes of antibacterial substances) [50]. Reference strains from the American Type Culture Collection (ATCC) *S. aureus* ATCC 43,300 and ATCC 25,923 were used to assess drug susceptibility.

### 4.3. Detection of Resistance Genes

The isolated strains were tested for the presence of genes encoding resistance to PEN (*bla*Z), methicillin (*mec*A and *mec*C), VAN (*van*A and *van*B), GEN (*aac(6′)-Ie-aph(2″)-Ia*), TET (*tet*K, *tet*L, and *tet*M), MLS_B_ (*erm*A, *erm*B, *erm*C, and *msr*A), and CHL *cat* (pC221), *cat* (pC194), and *cat* (pC223). The reaction primers and PCR conditions are presented in Table S1. The reactions were carried out in a T100 Thermal Cycler (Bio-Rad, Hercules, USA) using PCR Mix Plus (A&A Biotechnology, Gdańsk, Poland) and appropriate primers (Genomed S.A., Warsaw, Poland). *S. aureus* ATCC 43,300 was used as the reference strain.

### 4.4. MLST

MDR and VISA strains were selected for the MLST analysis. This technique was based on sequence analysis of 7 housekeeping genes: *arcC*, *aroE*, *glp*, *gmk*, *pta*, *tpi*, and *yqiL.* The primers used in this study are presented in Table S1. The PCR conditions were selected based on the protocol described by Enright et al. [45]. All reactions were carried out in a volume of 25 µL using Silver Taq MIX (Syngen Biotech, Wrocław, Poland) in a T100 Thermal Cycler (Bio-Rad, Hercules, USA). A Clean-up kit (A&A Biotechnology, Gdańsk, Poland) was used to purify the PCR products. DNA sequencing was performed with the Sanger method by Genomed S.A. (Warsaw, Poland). The obtained nucleotide sequences were compared with the alleles in the PubMLST database [51]. Based on allele combinations, STs and clonal complexes were assigned. The ST profiles were analyzed (minimum spanning tree method) with PHYLOViZ 2.0 software using the global optimal eBRUST algorithm. The nucleotide sequences of housekeeping genes were deposited in GenBank under the following accession numbers: *tpi*: OQ849231–OQ849345, *aroE*: OQ849346–OQ849460, *arcC*: OQ849461–OQ849575, *glpF*: OQ875252–OQ875366, *gmk*: OQ875367–OQ875481, *pta*: OQ875482–OQ875596, and *ygiL*: OQ875597–OQ875711.

### 4.5. ADSRRS-Fingerprinting

All *S. aureus* isolates were subjected to genotyping with the ADSRRS-fingerprinting technique according to the protocol described by Krawczyk et al. [52] with some modifications. First, genomic DNA was digested with 2 restriction enzymes: XbaI (10U) and BglII (5U) (Thermo Scientific, Waltham, USA) at 37 °C for 1 h, and then the ligation reaction was performed with T4 DNA ligase (Thermo Scientific, Waltham, USA) and appropriate adapters (25 °C for 1 h). After heat inactivation at 70 °C for 5 min, PCR was performed in a volume of 25 µL containing 1µL of ligation mix, Silver Taq MIX (Syngen Biotech, Wrocław, Poland), and 50 pmol of each primer (Genomed S.A., Warsaw, Poland). The reaction was carried out in a T100 Thermal Cycler (Bio-Rad, Hercules, USA) using the following thermal profile: 94 °C—5 min, 72 °C—5 min, 94 °C—5 min, 22 cycles (94 °C—30 s, 60 °C—30 s, and 72 °C—90 s), and 72 °C—7 min. Electrophoresis of PCR products was performed in 8% polyacrylamide gel and documented in GelDoc2000 (Bio-Rad, Hercules, USA). Similarity analysis of the electrophoretic profiles (UPGMA method) was performed using BIO-1D++ 11.9 software (Vilber, Lourmat, France) on the basis of the Jaccard correlation coefficient (tolerance and optimization 1%). Genotypes with 90% similarity were classified as one.

### 4.6. Statistical Analysis

Statistical analysis was performed using Statistica software 13.1 version (StatSoft, Poland). The Mann–Whitney U test was used to compare the occurrence of drug resistance and resistance genes of *S. aureus* isolates between the host groups (*p* < 0.05).

## Figures and Tables

**Table 1:** Occurrence of *S. aureus* in individual farms (number/percentage).

Host	A † (*n* = 35)	B (*n* = 19)	C (*n* = 30)	D (*n* = 10)	E (*n* = 29)	F (*n* = 26)	G (*n* = 31)	H (*n* = 5)	I (*n* = 22)	J (*n* = 30)	K (*n* = 25)	L (*n* = 1)	M (*n* = 25)	N (*n* = 32)	O (*n* = 32)	P (*n* = 36)	R (*n* = 27)	Total
Pigs (*n* = 248)														12/37.5	3/9	36/100	0	51/21
Cattle (*n* = 680)	1/3	1/5	1/3	0	12/41	3/11	4/13	3/60	1/4	0	8/32	1/100	1/4					36/5
Human carriers ‡ (*n* = 15)	nt §	1	2	2	0	0	1	nt	1	1	nt	nt	nt	0	nt	nt	2	10/67

† A–M—cattle farm; N–P—pig farm (number of swabs from animals). ‡ Owners of animals/animal keepers (number of swabs). § Not tested.

**Table 2:** Drug susceptibility of *S. aureus* isolated from different hosts (number/percentage of resistant isolates).

Host	Agent †														
	PEN	FOX *	VAN	GEN	TET *	ERY *	CLI * CLIind	QD	CIP	ENR	CHL	SXT	RIF	NIT	LZD
‡ H (140)	§ 96/69	6/4	25/18	13/9	24/17	49/35	26/19 7/5	3/2	26/19	13/9	7/5	2/1	1/1	3/2	1/1
P (51)	49/96	39/76	0/0	4/8	48/94	44/86	51/100 0/0	9/18	0/0	0/0	0/0	2/4	0/0	0/0	0/0
C (36)	3/8	1/3	5/14	0/0	2/6	1/3	1/3 0/0	0/0	2/6	1/3	0/0	0/0	0/0	0/0	0/0
W (12)	2/17	0/0	0/0	1/8	1/8	0/0	0/0 0/0	0/0	0/0	0/0	0/0	0/0	0/0	0/0	0/0
T (239)	150/63	46/19	30/13	18/8	75/31	94/39	78/33 7/3	12/5	28/12	14/6	7/3	4/2	1/0.4	3/1	1/0.4

† PEN—penicillin, FOX—cefoxitin (methicillin), VAN—vancomycin, GEN—gentamicin, TET—tetracycline, ERY—erythromycin, CLI—clindamycin, CLIind—inducible clindamycin resistance, QD—quinupristin-dalfopristin, CIP—ciprofloxacin, ENR—enrofloxacin, CHL—chloramphenicol, SXT—Trimethoprim-sulfamethoxazole, RIF—rifampin, NIT—nitrofurantoin, LZD—linezolid, ‡ strains isolated from (number): H—human, P—pigs, C—cattle, W—wildlife, T—total. § Number/percentage resistant strains; for vancomycin—the number/percent of strains with intermediate susceptibility to vancomycin (VISA, MIC = 4 µg/mL). * Statistically significant differences in the drug resistance of *S. aureus* strains between host group (Mann–Whitney U test, *p* < 0.05).

**Table 3:** Phenotypic drug resistance profiles of *S. aureus* isolated from different hosts.

Host	Resistance Profile †	Proportion (Number/Percentage)
human (*n* = 140) § ca (*n* = 10) ho (*n* = 130)	PEN	4/40 (B, I, J, G); 24/18
	CLI	2/1.5
	TET	1/0.8
	CHL	1/0.8
	CIP	2/1.5
	VAN	3/2
	PEN, VAN	1/10 (C), 11/8
	PEN, CLI	1/10(C)
	PEN, ERY	7/5
	PEN, TET	3/2
	PEN, SXT	1/0.8
	PEN, GEN	1/0.8
	PEN, CHL	1/0.8
	PEN, CIP	2/1.5
	CLI, ERY	3/2
	CIP, ENR	2/1.5
	TET, GEN	1/0.8
	TET, VAN	1/0.8
	ERY, CHL	1/0.8
	PEN, CLI, VAN	1/0.8
	PEN, CLI, ERY	4/3
	PEN, TET, ERY	2/20 (R), 3/2
	PEN, TET, CIP	1/0.8
	PEN, ERY, CIP	2/1.5
	PEN, CIP, VAN	1/0.8
	CLI, ERY, GEN	1/0.8
	CLI, ERY, VAN	1/0.8
	PEN, CLI, ERY, CIP	2/1.5
	PEN, CLI, ERY, GEN	2/1.5
	PEN, CLI, ERY, ENR	1/0.8
	PEN, CLI, TET, ERY	1/0.8
	PEN, FOX, CIP, ENR	1/0.8
	PEN, QD, TET, CIP	1/0.8
	PEN, ERY, CIP, VAN	1/0.8
	PEN, FOX, TET, CHL	1/0.8
	PEN, CLI, TET, ERY, VAN	1/10 (D)
	PEN, QD, TET, NIT, CIP,	1/0.8
	PEN, CLI, ERY, CIP, ENR	4/3
	PEN, CLI, TET, ERY, GEN	1/10 (D), 1/0.8
	PEN, FOX, CLI, ERY, CIP	1/0.8
	CLI, TET, ERY, CHL, CIP, ENR	1/0.8
	PEN, FOX, CLI, ERY, CIP, ENR	1/0.8
	PEN, CLI, TET, ERY, GEN, VAN	2/1.5
	PEN, ERY, GEN, CHL, CIP, ENR	1/0.8
	PEN, FOX, ERY, GEN, CIP, ENR, VAN	1/0.8
	CLI, SXT, TET, ERY, GEN, NIT, CHL, CIP, ENR	1/0.8
	PEN, FOX, CLI, QD, TET, RIF, ERY, GEN, NIT, LZD, VAN	1/0.8
	S	24/17
pigs (*n* = 51)	PEN, CLI	1/2 (N)
	PEN, CLI, TET	2/4 (N)
	CLI, QD, ERY	1/2 (N)
	PEN, CLI, TET, ERY	1/2 (N)
	PEN, CLI, TET, GEN	1/2 (N)
	PEN, CLI, QD, TET	3/6 (N)
	CLI, QD, ERY, GEN	1/2 (N)
	PEN, FOX, CLI, TET, ERY	36/71 (P)
	PEN, CLI, QD, TET, ERY	1/2 (N)
	PEN, FOX, CLI, QD, TET, ERY	1/2 (O)
	PEN, FOX, CLI, SXT, TET, ERY	1/2 (O)
	PEN, CLI, SXT, QD, TET, ERY, GEN	1/2 (N)
	PEN, FOX, CLI, QD, TET, ERY, GEN	1/2 (O)
cattle (*n* = 36)	PEN	2/6 (B, H)
	TET	1/3 (A)
	CIP	1/3 (E)
	VAN	5/14 (E (*n* = 2), F (*n* = 1), I (*n* = 1), M (*n* = 1)
	CLI, ERY	1/3 (G)
	PEN, FOX, TET, CIP, ENR	1/3 (L)
	S	25/69 (C (*n* = 1), E (*n* = 9), F (*n* = 2), G (*n* = 3), H (*n* = 2), K (*n* = 8))
wildlife (*n* = 12)	PEN	1/8
	GEN	1/8
	PEN, TET	1/8
	S	9/75

† PEN—penicillin, FOX—cefoxitin (methicillin), CLI—clindamycin, SXT—Trimethoprim-sulfamethoxazole, QD—quinupristin-dalfopristin, TET—tetracycline, RIF—rifampin, ERY—erythromycin, GEN—gentamicin, NIT—nitrofurantoin, LZD—linezolid, CHL—chloramphenicol, CIP—ciprofloxacin, ENR—enrofloxacin, VAN—vancomycin, S—susceptible. § ca—carrier (marked in blue); ho—hospitalized patient. (A–R)—farm markings. MDR strains are marked in red.

**Table 4:** Characterization of MDR strains (number/percentage).

Host †	Number of Drugs to Which Resistance Was Found							
	3	4	5	6	7	9	11	Total
H ca (*n* = 10)	2/20		2/20					4/40
H ho (*n* = 130)	14/11	10/8	7/5	5/4	1/0.8	1/0.8	1/0.8	39/30
P (*n* = 51)	3/6	6/12	37/73	2/4	2/4			50/98
C (*n* = 36)			1/3					1/3
W (*n* = 12)								
Total (*n* = 239)	19/8	16/7	47/20	7/3	3/1	1/0.4	1/0.4	94/39

† H ca—human carrier, H ho—human hospitalized patient, P—pigs, C—cattle, W—wildlife.

**Table 5:** Occurrence of resistance genes of *S. aureus* (number/percentage).

Host †	Resistance Genes											
	*bla*Z *	*mec*A *	*mec*C *	*aac(6′)-Ie-aph(2″)-Ia*	*tet*M *	*tet*K *	*erm*A *	*erm*B *	*erm*C	*msr*A	*Cat* (pC221)	*Cat* (pC223)
† H (140)	88/63	5/4	5/4	10/7	23/16	14/10	9/6	21/15	12/9	5/4	4/3	1/1
P (51)	49/96	39/76	39/76	1/2	47/92	39/76	36/71	39/76	3/6	0/0	0/0	0/0
C (36)	3/8	1/3	1/3	0/0	2/6	2/6	0/0	0/0	0/0	0/0	0/0	0/0
W (12)	1/8	0/0	0/0	0/0	1/8	1/8	0/0	0/0	0/0	0/0	0/0	0/0
T (239)	141/59	45/19	45/19	11/5	73/31	56/23	45/19	60/25	15/6	5/2	4/2	1/0.4

† Strains isolated from (number): H—human, P—pigs, C—cattle, W—wildlife, T—total. * Statistically significant differences in the drug resistance of *S. aureus* strains between host group (Mann–Whitney U test, *p* < 0.05).

**Table 6:** Frequency and distribution of antimicrobial resistance genes among *S. aureus* (number/percentage).

No. of Isolates Resistant to:	Number of Resistance Genes				
	0	1	2	3	4
penicillin (*n* = 150)	8/5	97/65: *bla*Z	1/0.7: *mec*A, *mec*C	44/29: *mec*A, *mec*C, *bla*Z	
cefoxitin (methicillin) (*n* = 46)	1/2		45/98: *mec*A, *mec*C		
gentamicin (*n* = 18)	7/39	11/61: *aac(6′)-Ie-aph(2″)-Ia*			
tetracycline (*n* = 75)	1/1	18/24: *tet*M	56/75: *tet*M, *tet*K		
erythromycin (*n* = 94)	21/22	25/27: *erm*B (14/15) *erm*C (10/11) *msr*A (1/1)	46/49: *erm*A, *erm*B (42/45) *erm*B, *erm*C (1/1) *erm*A, *erm*C (1/1) *erm*C, *msr*A (1/1) *erm*B, *msr*A (1/1)		2/2: *erm*A, *erm*B, *erm*C, *msr*A
clindamycin (*n*-85)	18/21	21/25: *erm*B (10/12) *erm*C (11/13)	44/52: *erm*A, *erm*B (42/49) *erm*B, *erm*C (1/1) *erm*A, *erm*C (1/1)	2/2: *erm*A, *erm*B, *erm*C	
quinupristin-dalfopristin (*n* = 12)	6/50	5/42: *erm*C (3/25) *erm*B (2/17)	1/8: *erm*B, *msr*A		
chloramphenicol (*n* = 7)	2/29	5/71: *cat* (pC221) (4/57) *cat* (pC223) (1/14)			

**Table 7:** Characteristics of VISA and MDR *S. aureus*.

CC ^a^	ST	*n*	Host ^b^	Genotypic Profile ^c^	Resistance Profile ^d^	Resistance Genes ^e^											
						*blaZ*	*mecA*	*mecC*	*aac*	*tetM*	*tetK*	*ermA*	*ermB*	*ermC*	*msrA*	cat221	cat223
1	1	1	human (ho)	XX	PEN, CLI, ERY, CIP	+								+			
1	9	1	pigs (N)	XVIII	PEN, CLI, TET	+											
1	9	3	pigs (N)	XVIII	PEN, CLI, QD, TET	+				+							
1	9	1	pigs (N)	XVIII	PEN, CLI, TET	+				+							
1	9	1	pigs (N)	XVIII	PEN, CLI, TET, ERY	+				+							
1	9	1	pigs (N)	XVIII	PEN, CLI, QD, TET, ERY	+				+							
1	9	1	pigs (N)	XVIII	PEN, CLI, TET, GEN	+				+							
1	2423	1	pigs (N)	XVIII	CLI, QD, ERY									+			
1	2423	1	pigs (N)	XVIII	CLI, QD, ERY, GEN									+			
5	5	1	human (ho)	XI	PEN, ERY, GEN, CIP, ENR, VAN	+			+						+		+
5	5	1	human (ho)	X	PEN, CLI, ERY, CIP, ENR								+				
5	5	1	human (ho)	XI	CLI, SXT, TET, ERY, GEN, NIT, CIP, CHL, ENR					+	+			+	+		
5	5	1	human (ho)	XI	VAN												
5	6	1	human (ho)	IX	PEN, ERY, CIP	+											
5	225	2	human (ho)	XI	PEN, CLI, ERY, CIP, ENR	+						+	+				
5	2750	1	human (ho)	XI	PEN, TET, FOX, CHL	+	+	+		+	+						
5	6158	1	human (ho)	XI	PEN, TET, ERY	+				+			+				
8	8	1	human (ho)	XIII	PEN, CLI, FOX, ERY, CIP, ENR	+	+	+						+			
8	8	1	human (ho)	XIV	PEN, TET, ERY, GEN, CIP, ENR, VAN	+	+	+	+								
8	8	1	human (ho)	XV	CLI, TET, ERY, CIP, CHL, ENR					+	+	+	+	+	+	+	
8	8	1	human (ho)	XIV	PEN, VAN	+											
15	14	1	human (ho)	II	PEN, ERY, CIP	+											
15	15	1	human (ho)	I	PEN, QD, TET, CIP	+				+	+						
15	15	1	human (ho)	I	PEN, QD, TET, NIT, CIP	+				+	+						
15	15	1	human (ho)	I	PEN, CLI, TET, ERY	+				+	+		+				
15	15	2	human (ho)	I	PEN, TET, ERY	+				+	+		+				
15	15	1	human (ho)	I	PEN, TET, CIP	+				+	+						
15	15	1	human (ho)	I	PEN, VAN	+											
15	582	1	human (ca, D)	I	PEN, CLI, TET, ERY, GEN	+				+			+	+			
15	582	2	human (ca, R)	I	PEN, TET, ERY	+				+							
15	582	1	human (ho)	II	PEN, VAN	+											
15	582	1	human (ho)	I	PEN, CLI, VAN	+											
15	8140	1	human (ho)	I	PEN, VAN	+											
22	22	1	human (ho)	XXIII	PEN, FOX, CIP, ENR	+	+	+									
22	217	1	human (ho)	XXIII	PEN, CLI, ERY	+											
22	217	1	human (ho)	XXIII	PEN, VAN	+											
30	30	1	human (ho)	XVI	PEN, CLI, ERY	+											
30	30	1	human (ho)	XVI	PEN, VAN	+											
30	34	1	human (ho)	XVI	PEN, VAN	+											
45	45	1	human (ca, D)	III	PEN, CLI, TET, ERY, VAN								+				
45	45	1	human (ho)	III	CLI, ERY, GEN								+				
45	45	1	human (ho)	III	PEN, CLI, ERY	+											
45	45	1	human (ho)	III	PEN, VAN	+											
45	45	1	human (ho)	III	CLI, ERY, VAN							+	+				
45	45	1	human (ho)	III	PEN, VAN	+											
45	8133	1	human (ho)	VIII	PEN, CLI, QD, TET, FOX, RIF, ERY, GEN, NIT, LZD, VAN	+			+	+			+		+		
45	8141	1	human (ho)	III	VAN												
97	97	1	human (ho)	XXVI	PEN, CIP, VEN	+											
97	97	1	human (ho)	XXVI	TET, VAN					+	+						
97	8137	1	human (ho)	XVII	PEN, VAN												
	7	1	human (ho)	XXV	PEN, CLI, TET, ERY, GEN, VAN	+			+	+				+			
	7	1	human (ho)	XXV	PEN, CLI, TET, ERY, GEN	+			+	+				+			
	7	1	human (ho)	XXV	PEN, CLI, ERY, GEN	+			+					+			
	7	1	human (ho)	XXV	PEN, CLI, TET, ERY, GEN, VAN	+			+	+		+		+			
	7	1	human (ho)	XXV	PEN, CIP, ERY, GEN	+			+								
	7	1	human (ho)	XXV	VAN												
	25	1	human (ho)	V	PEN, CLI, ERY, CIP	+											
	25	1	human (ho)	V	PEN, CLI, ERY, CIP, ENR	+											
	78	1	human (ho)	VII	PEN, ERY, CIP, VAN												
	133	1	cattle (M)	IV	VAN												
	182	1	human (ho)	XII	PEN, VAN	+											
	338	1	human (ho)	VI	PEN, CLI, ERY	+							+				
	479	1	cattle (I)	XXII	VAN												
	398	1	pigs (O)	XXVII	PEN, CLI, QD, TET, FOX, ERY	+	+	+	+	+	+		+				
	398	1	pigs (O)	XXVII	PEN, CLI, QD, TET, FOX, ERY, GEN	+	+	+		+	+		+				
	398	1	pigs (O)	XXVII	PEN, CLI, SXT, TET, FOX, ERY	+	+	+		+	+		+				
	398	36	pigs (P)	XXVII	PEN, CLI, TET, FOX, ERY	+	+	+		+	+	+	+				
	398	1	cattle (L)	XXVII	PEN, TET, FOX, CIP, ENR	+	+	+		+	+						
	8134	1	human (ho)	XXI	PEN, CLI, FOX, ERY, CIP		+	+						+			
	8135	1	pigs (N)	XIX	PEN, CLI, SXT, QD, TET, ERT, GEN	+				+				+			
	8136	1	human (ho)	XXIX	PEN, CLI, ERY, ENR								+				
	8138	1	human (ca, C)	XXIV	PEN, VAN	+											
	8139	1	cattle (F)	XXVIII	VAN	+											
	8139	2	cattle (E)	XXVIII	VAN												

^a^ Clonal complex. ^b^ ho—hospitalized patient; ca—carrier; N, D, R, M, I, O, P, L, N, C, F, and E—farm markings. ^c^ According to Figure 2. ^d^ PEN—penicillin, CLI—clindamycin, SXT—Trimethoprim-sulfamethoxazole, QD—quinupristin-dalfopristin, TET—tetracycline, FOX—cefoxitin (oxacillin), RIF—rifampin, ERY—erythromycin, GEN—gentamicin, NIT—nitrofurantoin, LZD—linezolid, CHL—chloramphenicol, CIP—ciprofloxacin, ENR—enrofloxacin, and VAN—vancomycin (VISA). ^e^ cat 221—cat (pC221), cat 223—cat (pC223), aac—aac(6′)-Ie-aph(2″)-Ia. STs recorded for the first time are marked in red.

**Table 8:** Material for research obtained from different hosts.

Host	Source	Number	Animal Farm Marking
human ho †	*S. aureus* strains from skin and soft tissue infections (collection of the Department of Pharmaceutical Microbiology, Medical University of Lublin)	130	-
human ca (*n* = 15)	nasal swabs	1	N
		2	R
		1	B
		2	C
		2	D
		1	E
		1	F
		1	G
		1	I
		1	J
		1	AJ
		1	AK
pigs (*n* = 248)	nasal swabs	32	N
		32	O
		36	P
		27	R
		30	S
		31	T
		30	U
		30	W
cattle (*n* = 680)	nasal swabs	35	A
		19	B
		30	C
		10	D
		29	E
		26	F
		31	G
		5	H
		22	I
		30	J
		25	K
		1	L
		25	M
		27	AA
		30	AB
		37	AC
		25	AD
		40	AF
		12	AG
		22	AH
		28	AI
		25	AJ
		67	AK
		33	AL
		46	AM
wildlife (*n* = 113): *Capreolus capreolus* and *Cervus elaphus*	nasal swabs		
		93	-
		20	-

† ho—hospitalized patient, ca—carrier (owners of animals/animals keepers).

## Data Availability

Data presented in this study are available from the corresponding author.

## Supplementary Materials

The following supporting information can be downloaded at: https://www.mdpi.com/article/10.3390/antibiotics12071137/s1, Table S1: Primers used in this study. References [45,53,54,55,56,57,58,59,60] are cited in the Supplementary Materials.
