# Supplementary material for: Multi-Host Pathogen Staphylococcus aureus—Epidemiology, Drug Resistance and Occurrence in Humans and Animals in Poland
Source: Antibiotics (Basel). 2023 Jun 30;12(7):1137. doi: 10.3390/antibiotics12071137 (PMC10376275; doi:10.3390/antibiotics12071137)
Supplement: Supplementary file 1 [file antibiotics-12-01137-s001.zip › antibiotics-2451176-supplementary.pdf]

Table S1. Primers used in this study.

Gene detected	Sequences of the primer (5′ to 3′)	Amplicon size (bp)	Reference
*mec*A	F: TCCAGATTACAACTTCACCAGG<br>R: CCACTTCATATCTTGTAACG	162	[1]
*mec*C	F: TCACCAGGTTCAACYCAAAA<br>R: CCTGAATCWGCTAATAATATTTC	356	[2]
*bla*Z	F: ACTTCAACACCTGCTGCTTTC<br>R: TGACCACTTTTATCAGCAACC	173	[3]
*erm*A	F: AAGCGGTAAACCCCTCTGA<br>R: TTCGCAAATCCCTTCTCAAC	190	[4]
*erm*C	F: AATCGTCAATTCCTGCATGT<br>R: TAATCGTGGAATACGGGTTTG	299	[4]
*msr*A	F: TCCAATCATTGCACAAAATC<br>R: AATTCCCTCTATTTGGTGGT	163	[3]
*tet*K	F: TTAGGTGAAGGGTTAGGTCC<br>R: GCAAACTCATTCCAGAAGCA	647	[5]
*tet*L	F: GTTGCGCGCTATATTCCAAA<br>R: TTAAGCAAACTCATTCCAGC	456	[5]
*tet*M	F: GTTAAATAGTGTTCTTGGAG<br>R: CTAAGATATGGCTCTAACAA	718	[5]
cat (pC221)	F: ATTTATGCAATTATGGAAGTTG<br>R: TGAAGCATGGTAACCATCAC	435	[6]
cat (pC194)	F: CGACTTTTAGTATAACCACAGA<br>R: GCCAGTCATTAGGCCTAT	570	[6]
cat (pC223)	F: GAATCAAATGCTAGTTTTAACTC<br>R: ACATGGTAACCATCACATAC	284	[6]
aac(6′)-Ie-aph(2″)-Ia	F: CCAAGAGCAATAAGGGCATA<br>R: CACTATCATACCACTACCG	220	[7]
*van*A	F: GGGAAAACGACAATTGC<br>R: GTACAATGCGGCCGTTA	732	[8]
*van*B	F: ACGGAATGGGAAGCCGA<br>R: TGCACCCGATTTCGTTC	647	[8]
*arc*C	F: TTG ATT CAC CAG CGC GTA TTG TC<br>R: AGG TAT CTG CTT CAA TCA GCG	456	[9]
*aro*E	F: ATC GGA AAT CCT ATT TCA CAT TC<br>R: GGT GTT GTA TTA ATA ACG ATA TC	456	[9]
*glp*F	F: CTA GGA ACT GCA ATC TTA ATC C<br>R: TGG TAA AAT CGC ATG TCC AAT TC	465	[9]
gmk	F: ATC GTT TTA TCG GGA CCA TC<br>R: TCA TTA ACT ACA ACG TAA TCG TA	429	[9]
pta	F: GTT AAA ATC GTA TTA CCT GAA GG<br>R: GAC CCT TTT GTT GAA AAG CTT AA	474	[9]
tpi	F: TCG TTC ATT CTG AAC GTC GTG AA<br>R: TTT GCA CCT TCT AAC AAT TGT AC	402	[9]
ygiL	F: CAG CAT ACA GGA CAC CTA TTG GC<br>R: CGT TGA GGA ATC GAT ACT GGA AC	516	[9]
